# Destruction of atherosclerotic plaque using pulse ultrasound with a planar rectangular transducer

**DOI:** 10.1186/2050-5736-3-S1-O72

**Published:** 2015-06-30

**Authors:** Christakis Damianou, Christos Christofi, Nicos Mylonas, Margarita Theodoulou, Christos Makarounas

**Affiliations:** 1 MEDSONIC, LTD, Limassol, Cyprus; 2 The Cyprus Institute, Limassol, Cyprus; 3 Frederick Research Center, Limassol, Cyprus; 4 Cyprus University of Technology, Limassol, Cyprus

## Background/introduction

The aim of this paper is to present a feasibility study for using mechanical mode ultrasound for destroying atherosclerotic plaque with MRI monitoring.

## Methods

An MRI safe flat rectangular (3x10 mm2) transducer operating at 5 MHz was used. A spatial average temporal average intensity of 10 W/cm2 for 120 s was used, with DF of 10 % and 1 ms pulse repetition period. This optimized protocol was applied in a rabbit model. The plaque in the rabbit was created using a 2 % cholesterol diet.

## Results and conclusions

During the course of the diet, the aorta of the rabbit was imaged using high resolution T2 W FSE every one month in order to assess the progress of the growth of the plaque. Selected rabbits were sacrificed in one, two, and three months since the initiation of the diet in order to assess the growth of the plaque histologically. Approximately 50 % of the artery was covered by plaque in 3 months since the initiation of the diet. This was confirmed using MRI and histology. The proposed mechanical protocol has successfully destroyed plaque in the aorta of the rabbit. The size of the rabbit aorta has similar size with the coronary or carotid arteries in humans. The rabbit is a good model for creating atherosclerotic plaque.

